# Mesalamine-Induced Myocarditis

**DOI:** 10.4061/2010/930190

**Published:** 2010-09-13

**Authors:** Olivier Merceron, Clement Bailly, Antoine Khalil, Florence Pontnau, Nadjib Hammoudi, Richard Dorent, Pierre-Louis Michel

**Affiliations:** ^1^Cardiology Department, Tenon University and Medical School, Assistance Publique-Hôpitaux de Paris, 4, rue de la Chine, 75020 Paris, France; ^2^Pierre et Marie Curie University, Paris, France; ^3^Radiology Department, Tenon University and Medical School, Assistance Publique-Hôpitaux de Paris, 4, rue de la Chine, 75020 Paris, France

## Abstract

Nowadays mesalamine is a common treatment for Crohn's disease and hypersensitive reactions to this product have been reported. Yet there is limited information concerning mesalamine-induced myocarditis and its mechanism is not known. We described a case of mesalamine-induced myocarditis in Crohn's disease of the colon.

## 1. Introduction

Mesalamine is a well-known treatment for inflammatory bowel disease and drug reactions to this product are uncommon. Although rare, myocarditis attributed to mesalamine has been reported but the mechanism by which mesalamine might prompt myocardial inflammation is not clearly identify. We report a case of mesalamine-induced myocarditis in Crohn's disease of the colon.

## 2. Case Report

**Figure 1:**
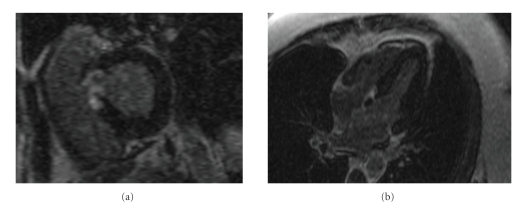
Cardiac MR shows on the late enhancement sequence, transmural enhancement of the basal interventricular septum ((a) short axis, (b) long axis).

A 41-year-old female with Crohn's disease on chronic treatment with mesalamine for several years was admitted to our hospital with severe “anginal” chest pain. The Crohn's disease course was well controlled with no exacerbation for the past two years. The patient had no personal and familial history of cardiac abnormality or dysfunction, no hypertension or diabetes, no known allergies, and no other home medication. There was no reported infection in the last 6 months. Physical examination was normal and there was no sign of cardiac failure. The chest radiograph on admission was normal. Initial electrocardiogram showed sinus rhythm, a first-degree AV block, and flattened T-waves associated with increased Q-waves in the D3 lead. Laboratory tests revealed an elevated cardiac troponin I of 6,2 *μ*g/L (peak concentration of cardiac troponin I of 8,35 *μ*g/L), a Creatin kinase (CK) concentration of 258 U/L, a C-reactive protein (CRP) concentration of 8 mg/dL, and others blood tests within normal parameters (including leucocytes and eosinophils concentrations). An echocardiogram showed a mildly dilated left atrium and limited hypokinesis of the apical half of the left ventricle's inferior wall. The estimated ejection fraction was normal at 65%. Coronary angiography was performed demonstrating normal epicardial vessels. Cardiac magnetic resonance imaging (MRI) with delayed enhancement showed epicardial enhancement in the apical inferior wall and transmural enhancement in the interventricular septum (Figures 1(a) and 1(b)). There was no sign of myocardial infarction and thus, the patient was diagnosed with acute myocarditis.

**Figure 2:**
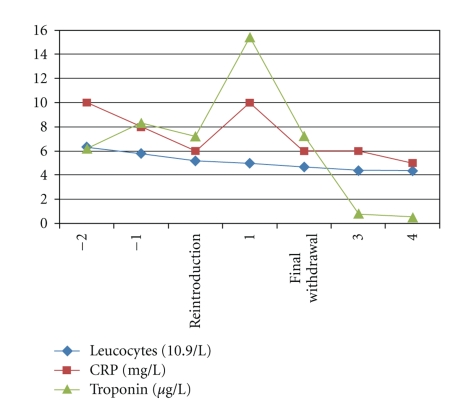
Course of systemic inflammation and troponin after mesalamine re-introduction in a patient with mesalamine induced myocarditis in Crohn's disease of the colon.

On the patient's admission, mesalamine was discontinued and two days after this withdrawal, the chest pain was gone and cardiac troponin I (7,73 *μ*g/L) and CK decreased to normal levels. As mesalamine has not first been considered as a potential agent, it was administered once more. The reintroduction of mesalamine induced a reapparition of the symptoms with an increased cardiac troponin I concentration (15,44 *μ*g/L) (Figure 2). The symptoms resolved without any treatment following a final withdrawal of mesalamine. The patient has done well since discharge. No other cardiac MRI was performed.

## 3. Discussion

Cardiac diseases can be associated with Crohn's disease as extraintestinal manifestation of the Inflammatory Bowel Disease or as a consequence of drug-induced side effects. Mesalamine-induced myocarditis is a rare but potentially serious occurrence and several cases were described in the literature [1–4], generally during the first weeks of treatment but on occasion, following treatment extending over years [5]. Yet, the exact mechanism by which mesalamine might prompt myocardial inflammation is not clearly identified. Rarely, mesalamine can induce hypersensitive reactions such as, hypersensitivity pneumonitis, angioedema, skin rashes, hypereosinophilia and thus, a mechanism of hypersensitivity to the drug rather than a direct cytotoxic effect is suspected [1–6]. The diagnosis of hypersensitivity myocarditis (HSM) is also suggested by the fact that, in all of the cases identified as being the result of mesalamine toxicity [1–3], there had been a clear improvement following the discontinuation of the drug. An eosinophilic infiltration of the myocardium on endomyocardial biopsy has been described by Stelts et al. [3] which seems to confirm the link between mesalamine and HSM. 

In our case, the clinical and biological evolution was considered as atypical for a viral myocarditis and the diagnosis of HSM was first evoked on the late gadolinium enhancement in the interventricular septum. This MRI feature has previously been described in multisystem disorders with hypersensitivity reaction such as, sarcoidosis. Moreover, this is the first time in the literature that a case has been described with a provocation test, carried out by administering the potential etiologic agent once more; as in the reports reviewed [2, 5], the severe clinical status of the patients made this study hazardous. In our case, this test was carried out unintentionally but it makes the hypersensitive reaction responsible of mesalamine-induced myocarditis indisputable. For this reason, no myocardial biopsy has been taken and no viral serum antibody titers were made but also due to the lack of official recommendation and the difficulty of viral isolation. Different mechanisms could explain HSM but the exact one has not yet been identified. The most probable hypothesis seems to be an inhibition of cyclooxygenase-1 (COX1) by the drug, which may accelerate the metabolism of arachidonic acid toward lipoxygenase products such as, leukotrienes [6]. This overproduction of leukotriene metabolites may induce proinflammatory signaling, initiating the hypersensitive reaction by liberating eosinophil-stimulating cytokines, and will lead to the myocarditis. Further examination methods such as, immunohistochemical or molecular biological techniques have not been established so far. 

However, this report shows that the risk of mesalamine-induced myocarditis may be underestimated and that the possibility of cardiac involvement needs to be considered in patients with Inflammatory Bowel Disease on chronic treatment with mesalamine and chest pain. In those cases, the majority of patients recover in a few days after withdrawal of the causative agent.
